# Resveratrol regulates type II collagen and COX-2 expression via the ERK, p38 and Akt signaling pathways in rabbit articular chondrocytes

**DOI:** 10.3892/etm.2014.1484

**Published:** 2014-01-10

**Authors:** SEONG-HUI EO, HONG-SIK CHO, SONG-JA KIM

**Affiliations:** 1 Department of Biological Sciences, College of Natural Sciences, Kongju National University, Gongju, Chungnam 314-701, Republic of Korea; 2 The University of Tennessee Health Science Center, Memphis, TN 38163, USA; 3 Department of Orthopaedic Surgery, Campbell Clinic, Memphis, TN 38163, USA; 4 Veterans Affairs Medical Center, Memphis, TN 38163, USA

**Keywords:** chondrocytes, resveratrol, type II collagen, cyclooxygenase-2

## Abstract

Resveratrol, a naturally occurring polyphenolic phytoalexin antioxidant compound present in grapes and red wine, has been reported to induce various biochemical responses. It has been shown to possess anti-aging, anti-inflammatory and anti-proliferative activities in several cell types. However, the effects of resveratrol in normal cells, including chondrocytes, have not yet been clearly elucidated. The aim of the present study was to evaluate the effects of resveratrol on differentiation and inflammation in rabbit articular chondrocytes and to investigate the underlying mechanism of action. Rabbit articular chondrocytes were treated with 20 μM resveratrol for different time periods or with various concentrations of resveratrol for 24 h. It was observed that the expression levels of type II collagen and sulfated proteoglycan, as determined by western blot analysis and Alcian blue staining, respectively, increased following treatment with resveratrol in a concentration-dependent manner at concentrations up to 20 μM and then decreased at higher concentrations. The expression levels of cyclooxygenase (COX-2) and prostaglandin E_2_ (PGE_2_) began to increase at 10 min after the addition of resveratrol, reached peak levels at 3 h and decreased from the peak level thereafter, as determined by western blot analysis and PGE_2_ assay, respectively. It was also demonstrated that resveratrol caused phosphorylation of mitogen-activated protein kinase proteins [extracellular signal-regulated kinases (ERK), p38 and c-Jun N-terminal kinases (JNK)] and Akt in rabbit articular chondrocytes. The inhibition of ERK, p38 kinase, phosphoinositide 3-kinase (PI3K) and Akt with PD98059, SB203580, LY294002 and triciribine, respectively, suppressed resveratrol-induced type II collagen and COX-2 expression. However, inhibition of JNK with SP600125 produced no clear changes in the expression levels of type II collagen and COX-2. The results suggest that resveratrol in articular chondrocytes stimulates differentiation and inflammation via the ERK, p38 and Akt signaling pathways.

## Introduction

Chondrocytes in articular cartilage are differentiated from mesenchymal cells during embryonic development (1–3). The differentiated chondrocytes are able to proliferate and undergo hypertrophic maturation. Cartilage is composed of a dense extracellular matrix, made up from macromolecules such as type II collagen, sulfated proteoglycan and fibronectin (4). This biosynthetic composition of chondrocytes is maintained during complex biological processes, including cartilage development, differentiation and repair. However, the differentiated chondrocyte phenotype is unstable in culture and destroyed in degenerative diseases, such as osteoarthritis (OA) and rheumatoid arthritis (RA) (5–9).

Cyclooxygenase (COX) is an enzyme that catalyzes the conversion of arachidonic acid to prostaglandin H_2_, the precursor of a variety of biologically active mediators such as prostaglandin E_2_ (PGE_2_), prostacyclin and thromboxane A2 (10,11). Two isoforms of COX are COX-1 and COX-2. COX-1 is constitutively expressed in a wide variety of tissues, is ubiquitous in its distribution, and is thought to be involved in tissue homeostasis and maintenance of the levels of prostaglandins. COX-2 is an enzyme induced by pro-inflammatory cytokines, tumor promoters, oncogenes and growth factors, and is involved mainly in the regulation of inflammatory responses in numerous types of cell, such as monocytes, fibroblasts and endothelial cells (2,12).

Resveratrol (C_14_H_12_O_3_; 3,5,4′-trihydroxy-*trans*-stilbene) was first identified in the roots of white hellebore (*Veratrum grandiflorum*) in 1940 (13). Resveratrol is a natural polyphenolic compound that is also found in the skin of red grapes, cranberries and peanuts, and the root extracts of the weed *Polygonum cuspidatum* (14–16). Numerous signaling pathways involving resveratrol have been evaluated and a number of its targets and mechanisms of action have been identified. It has been reported that resveratrol has antitumor activity and immunomodulatory, antioxidative and anti-inflammatory functions, as well as numerous biological activities. Resveratrol has been shown to exhibit *in vitro* as well as *in vivo* chemopreventive and chemotherapeutic activities (15,17–20).

Elmali *et al* observed a significant protective effect of resveratrol injections on articular cartilage degradation in rabbit models for OA and RA via histological analysis *in vivo* (21). Resveratrol has been demonstrated to suppress aging by activating the SIRT1 gene, which suppresses cell apoptosis (22–26). In human articular chondrocytes, Czaki *et al* elucidated anti-apoptotic and anti-inflammatory regulatory mechanisms mediated by resveratrol (27). In human articular chondrocytes, resveratrol together with curcumin was shown to suppress the apoptosis induced by IL-1β through stimulation of the mitogen-activated protein kinase (MAPK) signaling pathway (27).

However, the effects of resveratrol on differentiation and the inflammatory response in normal cells, including chondrocytes, and the mechanism by which resveratrol acts are not clearly understood. As a result, the present study was conducted to investigate the effects of resveratrol on differentiation and the inflammatory response of rabbit chondrocytes and to analyze the subsequently regulated intracellular signal transduction pathways.

## Materials and methods

### Reagents and antibodies

Resveratrol was purchased from Sigma-Aldrich (St. Louis, MO, USA). The resveratrol was diluted in sterile dimethylsulfoxide (Sigma-Aldrich; final concentration in the medium was >1%) and stored at −20°C. Dulbecco’s modified Eagle’s medium (DMEM) and fetal bovine serum (FBS) were purchased from Invitrogen (Burlington, ON, Canada). Streptomycin, penicillin and SP600125 were obtained from Sigma-Aldrich. SB203580 (SB), PD98059 (PD), LY294002 (LY) and triciribine (TB) were purchased from Calbiochem (San Diego, CA, USA). Type II collagen, actin, COX-2 and pERK antibodies were obtained from Santa Cruz Biotechnology Inc. (Santa Cruz, CA, USA) and pAkt, p38 and pJNK were from Cell Signaling Technology (Danvers, MA, USA).

### Cell culture

Rabbit articular chondrocytes were isolated from the cartilage of two-week-old New Zealand white rabbits (KOATECH, Pyeongtaek-si, Gyeonggi-do, Korea) using enzymatic digestion, as described previously (28). The cartilage slices were dissociated enzymatically for 6 h in 0.2% collagenase type II (381 U/mg solid; Sigma-Aldrich) in DMEM. Following collection of individual cells by brief centrifugation at 230 × g for 10 min and 20°C, the cells were suspended in DMEM supplemented with 10% (v/v) FBS, 50 μg/ml streptomycin and 50 U/ml penicillin. The cells were then plated on culture dishes at a density of 5×10^4^ cells/cm^2^. The medium was changed every two days, and the cells reached confluence after approximately five days. After three days the cell cultures were treated with resveratrol. The following pharmacological agents were added 1 h prior to the addition of resveratrol: SB to inhibit p38 kinase, PD to inhibit ERK, and LY and TB to inhibit phosphoinositide 3-kinase (PI3K) and Akt, respectively. The study was approved by the Ethics Committee of Kongju National University (Gongju, Republic of Korea; IRB no. 2011–2).

### Western blot analysis

Whole cell lysates were prepared by extracting proteins using a cold radioimmunoprecipitation assay buffer [50 mM Tris-HCl, pH 7.4; 150 mM NaCl; 1% Nonidet P-40; and 0.1% sodium dodecylsulfate (SDS); supplemented with protease inhibitors (10 μg/ml leupeptin, 10 μg/ml pepstatin A, 10 μg/ml aprotinin and 1 mM 4-(2-aminoethyl)benzenesulfonyl fluoride and phosphatase inhibitors (1 mM NaF and 1 mM Na_3_VO_4_)] obtained from Sigma-Aldrich. The lysates were size-fractionated by SDS-polyacrylamide gel electrophoresis and transferred to a nitrocellulose (NC) membrane (Whatman Schleicher and Schuell, Dassel, Germany). The NC sheet was then blocked with 5% non-fat dry milk in Tris-buffered saline. Antibodies against type II collagen, COX-2, pp38, pERK, pJNK and Akt were used for probing corresponding NC blots overnight at 4°C. The membranes were then washed three times with Tris-buffered saline/Tween-20 and incubated with horseradish peroxidase-conjugated secondary antibody (Sigma-Aldrich) for 2 h followed by exposure in an LAS-4000 imager (Fujifilm Corp., Tokyo, Japan) according to the manufacturer’s instructions. The experimental results were transformed into in numerical values using Image J 1.41 (Software Inquiry, Quebec, Canada).

### PGE_2_ assay

PGE_2_ production in the articular chondrocytes was determined by measuring the levels of cellular and secreted PGE_2_ with an assay kit purchased from Assay Design Inc. (Ann Arbor, MI, USA). A PGE_2_ linked immunosorbent assay kit was purchased from Amersham Pharmacia Biotech (Piscataway, NJ, USA). Briefly, the chondrocytes were seeded in standard 96-well microtiter plates at a density of 2×10^4^ cells/well and treated with various reagents, such as resveratrol, for 1 h prior to treatment with SB, PD, LY and TB for 3 h after treatment with resveratrol in the absence or presence of inhibitors (SB, PD, LY and TB). The amount of PGE_2_ present in total cell lysates was quantified according to the kit manufacturer’s instructions. Levels were calculated against a standard curve of PGE_2_.

### Immunofluorescence staining

The expression levels and distribution of type II collagen and COX-2 in the rabbit articular chondrocytes were determined by indirect immunofluorescence microscopy, as described previously (28). Rabbit chondrocytes were fixed with 3.5% paraformaldehyde in phosphate-buffered saline (PBS) for 15 min at room temperature, and permeabilized and blocked with 0.1% Triton X-100 and 5% fetal calf serum in PBS for 30 min. The fixed cells were washed three times with PBS and incubated for 2 h with antibodies against type II collagen (Santa Cruz Biotechnology, Inc.) or COX-2 (Cayman Chemical, Ann Arbor, MI, USA). The cells were washed and incubated with rhodamine or fluorescein-conjugated secondary antibodies for 1 h, washed with PBS, and observed under a fluorescence microscope (Olympus, Tokyo, Japan).

### Immunohistochemical staining

The cartilage explants (125 mm^3^) were fixed in 4% paraformaldehyde in PBS for 24 h at 4°C, washed with PBS, dehydrated with graded ethanol, embedded in paraffin and sectioned into 4-μm slices as described previously (28). The sections were stained by the standard procedures using antibodies against type II collagen or COX-2, and visualized by development with an EnVisionTM+ kit purchased from Dako (Carpinteria, CA, USA) following the manufacturer’s instructions.

### Determination of chondrocyte phenotype

The cells were fixed with 95% methanol at −20°C for 2 min and stained with 0.1% Alcian blue (Sigma Aldrich) in 0.1 M HCl overnight. The chondrocytes were washed three time with PBS buffer and 6 M guanidine HCl was added for 6 h. Production of sulfated proteoglycan was measured at 620 nm by an enzyme-linked immunosorbent assay.

### Data analysis and statistics

The results are expressed as the mean ± standard deviation. The values were calculated from the specified number of determinations. The significance of the differences between the experimental and control groups was assessed by one-way ANOVA. A value of P<0.05 was considered to indicate a statistically significant difference.

## Results

### Resveratrol regulates the differentiation of chondrocytes

**Figure 1:**
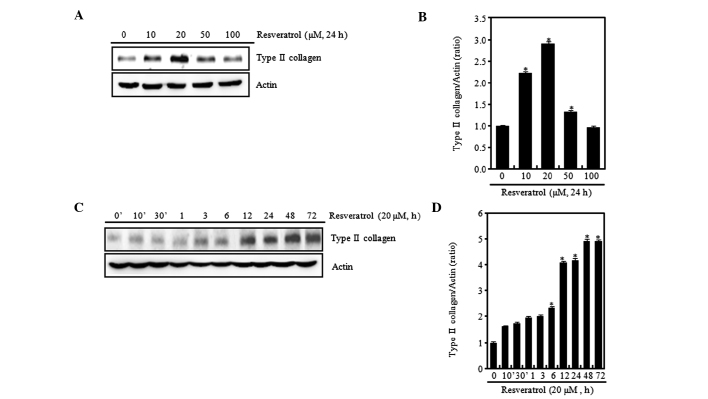
Resveratrol regulates type II collagen expression in rabbit articular chondrocytes. Rabbit articular chondrocytes were treated with various concentrations of resveratrol for the indicated time periods. (A and C) The expression levels of type II collagen were analyzed by western blot analysis. Actin was used as a loading control. (B and D) The relative amounts of type II collagen were quantified by densitometric measurement (ImageJ). The data represent a typical experiment, whereby similar results were obtained from three experiments. ^*^P<0.05, compared with untreated cells.

**Figure 2:**
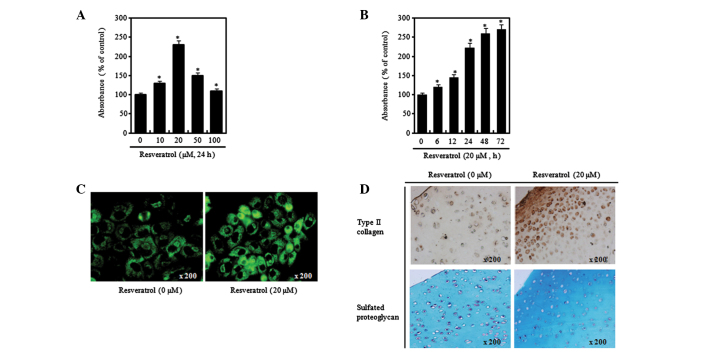
Resveratrol (Res) regulates differentiation in rabbit articular chondrocytes. Rabbit articular chondrocytes were untreated or treated (A) with various concentrations of resveratrol for 24 h or (B) with 20 μM resveratrol for the specified time periods. (C) Expression of type II collagen was determined by immunofluorescence staining and (D, upper panel) immunohistochemical staining (magnification, ×200). (D, lower panel) Sulfated proteoglycan was detected in tissue by Alcian blue staining. The data represent a typical experiment, whereby similar results were obtained from four experiments. ^*^P<0.05, compared with untreated cells.

An aim of this study was to determine whether resveratrol regulates the expression of type II collagen and sulfated proteoglycan in rabbit articular chondrocytes (Fig. 1). Various concentrations of resveratrol were tested, and at low concentrations (≤20 μM) it was found that the expression levels of type II collagen gradually increased in a concentration-dependent manner when compared with those in the control chondrocytes without resveratrol treatment. However, at a high concentration (100 μM), the expression level of type II collagen was observed to be reduced (Fig. 1A). When cells were treated with 20 μM resveratrol, type II collagen expression levels were increased in a time-dependent manner (Fig. 1C). The expression levels were determined using ImageJ software (Fig. 1B and D). Alcian blue staining was used to identify the levels of sulfated proteoglycan, which is an extracellular substrate molecule commonly used as another marker protein for differentiation of chondrocytes. Similar to type II collagen expression, the Alcian blue staining results showed that the levels of proteoglycan increased compared with those in the control when the chondrocytes were treated with 20 μM resveratrol, while they were less elevated when the chondrocytes were treated with 100 μM resveratrol (Fig. 2A). Moreover, it was observed that the changes in the proteoglycan levels in relation to time also started to increase from 24 h after the addition of resveratrol, similar to the effect on the expression of type II collagen (Fig. 2B). In order to verify the aforementioned results at chondrocytic cellular and tissue levels, immunofluorescence and immunohistochemical staining assays were performed to identify the levels of type II collagen and proteoglycan. As a result of performing immunofluorescence staining using type II collagen antibody following treatment with 20 μM resveratrol for 24 h, increased expression levels of type II collagen in resveratrol-treated cells were confirmed (Fig. 2C). Moreover, at the cartilaginous tissue level, increased expression levels of type II collagen were confirmed (Fig. 2D, upper panel). In addition, the results of performing Alcian blue staining following treatment of the cartilaginous tissue with 20 μM resveratrol for 24 h showed increased levels of proteoglycan in the resveratrol-treated tissues (Fig. 2D, lower panel). In general reference to the aforementioned results, it was found that chondrocytic differentiation was regulated differentially according to the concentration of resveratrol, and inducement of chondrocytic differentiation was identified after 24 h of treatment with resveratrol at a low concentration (20 μM).

### Resveratrol induces an inflammatory response in chondrocytes

**Figure 3:**
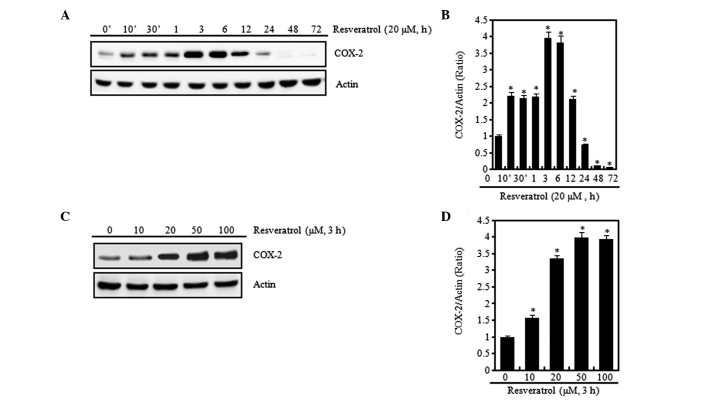
Resveratrol regulates the expression of COX-2 in rabbit articular chondrocytes. Rabbit articular chondrocytes were treated (A and B) with 20 μM resveratrol for 0–72 h or (C and D) with 10–100 μM Res for 3 h. (A and C) Expression levels of COX-2 were determined by western blot analysis. Actin was used as a loading control. (B and D) The relative amounts of COX-2 were quantified by densitometric measurement (with ImageJ software). The data represent a typical experiment, whereby similar results were obtained from three experiments. ^*^P<0.05, compared with untreated cells. COX-2, cyclooxygenase-2.

**Figure 4:**
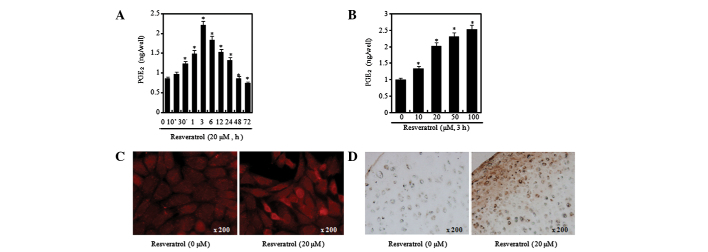
Resveratrol (Res) regulates the inflammatory response in rabbit articular chondrocytes. (A) Rabbit articular chondrocytes were untreated or treated with 20 μM resveratrol for the indicated time periods. (B) Rabbit articular chondrocytes were untreated or treated with the indicated concentrations of resveratrol for 3 h. PGE_2_ production was measured using a PGE_2_ assay kit. (C) Rabbit articular chondrocytes or (D) cartilage explants were untreated or treated with 20 μM resveratrol for 24 h. Expression of COX-2 was detected by immunofluorescence staining. The data represent a typical experiment, whereby similar results were obtained from three experiments. ^*^P<0.05, compared with untreated cells. PGE_2_, prostaglandin E_2_.

Previous studies have shown that the expression of COX-2 increases the levels of PGE_2_ and that PGE_2_ induces various inflammatory reactions (10,29). In the present study, chondrocytes were treated with resveratrol at 20 μM for different time periods (Fig. 3A and B) or with various concentrations of resveratrol for 3 h (Fig. 3C and D). Stimulation of cells with resveratrol induced a marked increase in COX-2 expression levels, which was apparent within 3 h after treatment with resveratrol. The COX-2 expression levels peaked at 3 h and the subsequent reduction was detectable for up to 72 h (Fig. 3A and B). Concentration-dependent increases in COX-2 expression levels were measured by western blot analysis and densitometric analysis (Fig. 3C and D). In order to find out more clearly whether or not resveratrol induces an inflammatory response in chondrocytes, a PGE_2_ assay was performed to evaluate the levels of PGE_2_, which is a product of COX-2, and changes in the expression levels of COX-2 at chondrocytic cellular and tissue levels were identified through immunofluorescence and immunohistochemical staining (Fig. 4). As a result of performing the PGE_2_ assay following the treatment of chondrocytes with resveratrol at different concentrations and time periods, it was possible to verify that the levels of PGE_2_ greatly increased at 3 h after treatment with 20 μM resveratrol but had begun to decrease by 6 h, in a similar manner to COX-2 (Fig. 4A). Moreover, it was confirmed that the PGE_2_ production levels were increased by resveratrol in a concentration-dependent manner (Fig. 4B). In order to identify the levels of COX-2 expression at the chondrocytic cell and tissue levels, immunofluorescence and immunohistochemical staining was conducted. Increased COX-2 expression levels were observed in the cells and tissues treated with resveratrol (Fig. 4C and D). The aforementioned results signify that resveratrol induces an inflammatory response in chondrocytes.

### Resveratrol increases the activation of MAPK and Akt

**Figure 5:**
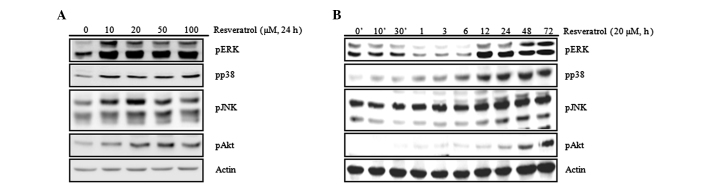
Resveratrol induces the activities of MAPKs and pAkt in rabbit articular chondrocytes. Rabbit articular chondrocytes were untreated or treated (A) with the indicated concentrations of resveratrol for 24 h, or (B) with 20 μM resveratrol for the indicated time periods. Expression of the MAPK proteins (pERK, pp38 and pJNK) and pAkt was determined by western blot analysis. Expression of actin was used as a loading control. The data represent the results of a typical experiment from at least four independent experiments. MAPK, mitogen-activated protein kinase; ERK, extracellular signal-regulated kinase; JNK, phosphorylated c-Jun N-terminal kinase.

**Figure 6:**
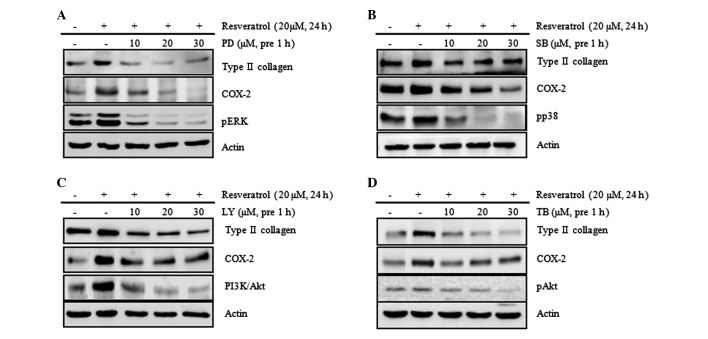
Resveratrol induces type II collagen and COX-2 expression via the ERK, p38 and Akt signaling pathways in rabbit articular chondrocytes. Rabbit articular chondrocytes were untreated or treated with the indicated concentrations of the inhibitors: (A) PD, an inhibitor of ERK; (B) SB, an inhibitor of p38; (C) LY, an inhibitor of PI3K/Akt; or (D) TB, an inhibitor of Akt for 1 h and then treated with 20 μM resveratrol for 24 h. Expression of pERK, pp38, pAkt, type II collagen and COX-2 was detected by western blot analysis. Expression of actin was used as the loading control. The data represent the results of a typical experiment from at least four independent experiments. PD, PD98059; SB, SB203580; LY, LY294002; TB, triciribine; COX-2, cyclooxygenase-2; ERK, extracellular signal-regulated kinase; PI3K, phosphoinositide 3-kinase.

**Figure 7:**
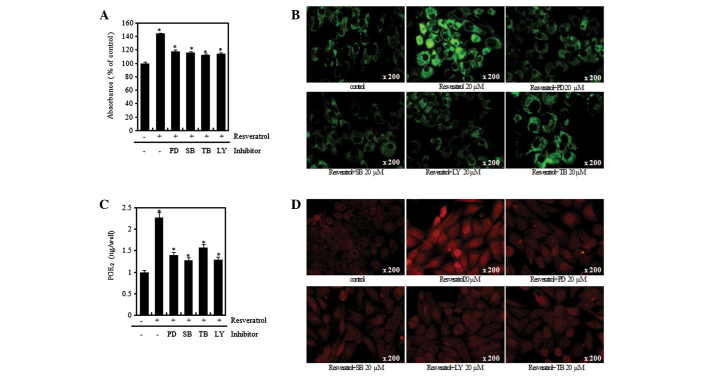
Resveratrol (Res) induces differentiation and inflammation in rabbit articular chondrocytes. (A) Rabbit articular chondrocytes were untreated or treated with the 20 μM of inhibitors (PD98059, SB203580, LY294002) and triciribine (TB) for 1 h and then treated with 20 μM Res for 24 h. Accumulation of sulfated proteoglycan was determined by Alcian blue staining. Expression of (B) type II collagen and (D) COX-2 was determined by immunofluorescence staining (magnification, ×200). (C) Rabbit articular chondrocytes were untreated or treated with the 20 μM of inhibitors (PD98059, SB203580, LY294002) and triciribine (TB) for 1 h and then treated with 20 μM Res for 3 h. ^*^P<0.05, compared with untreated cells. PD, PD98059; SB, SB203580; LY, LY294002; TB, triciribine; COX-2, cyclooxygenase-2; PGE_2_, prostaglandin E_2_.

To investigate through which signal transduction system the chondrocytic differentiation and inflammatory response due to resveratrol are regulated, the activation of MAPK and PI3K was examined. The MAPK and PI3K signal transduction pathways are closely associated with the regulation of chondrocytic differentiation and the inflammatory response (Fig. 5). The results confirmed an increase in activation of MAPK-related proteins (ERK, p38 and JNK) and Akt due to resveratrol (Fig. 5A). To study the changes in the expression levels of the proteins associated with cellular signal transduction according to treatment time, western blot analysis was performed following treatment with 20 μM of resveratrol for various time periods up to 72 h. The results showed that the activation levels of MAPK-related proteins (ERK, p38 and JNK) started to increase from 6 h and those of Akt started to increase from 24 h after the resveratrol treatment (Fig. 5B). Such results signify that chondrocytic differentiation and the inflammatory response induced by resveratrol are associated with activation of MAPK and Akt. Accordingly, inhibitors of MAPK-related proteins (ERK inhibitor, PD; p38 inhibitor, SB; JNK inhibitor, SP), TB (an inhibitor of Akt), and LY (an inhibitor of PI3K; the PI3K pathway is an upstream signal transduction pathway for Akt), were used to clearly identify the signal transduction pathways that are regulated by resveratrol. The chondrocytes first underwent pretreatment 1 h prior to resveratrol treatment to block the ERK, p38, JNK and Akt signaling pathways and were then treated with resveratrol, following which changes to chondrocytic differentiation and the inflammatory response proteins were studied through western blot analysis, immunofluorescence staining, Alcian blue staining and PGE_2_ assay (Fig. 6 and 7). The results of these assays showed that the resveratrol-induced increases in the expression levels of type II collagen and COX-2 were attenuated by treatment with SB, PD, LY and TB (Fig. 6). However, no changes to the levels of type II collagen and COX-2 expression due to SP treatment were observed (data not shown). Following verification using Alcian blue staining, immunofluorescence staining and PGE_2_ assay methods, the resveratrol-induced increases in the levels of proteoglycan and expression levels of type II collagen and COX-2 were observed to be similarly attenuated by PD, SB, TB and LY treatment (Fig. 7). Such results indicate that the differentiation and inflammatory response induced by resveratrol are mediated through the ERK, p38 and Akt signal transduction pathways.

## Discussion

Resveratrol, one of the major stilbenes, has a structure that is related to the synthetic estrogen diethylstilbestrol. It comprises two phenol rings linked by a styrene double bond and exists in two isoforms (19,30). Resveratrol has been demonstrated to process anticancer, anti-aging, anti-inflammatory and neuroprotective activities (31). Resveratrol has also been found to exhibit diverse biological effects; it induces MMP-9 expression and cell migration via the p38 kinase and PI3K pathway in HT1080 human fibrosarcoma cells (32) and induces differentiation via reduction of the expression of MMPs; this regulation is mediated by the p38 and JNK pathway in HTB94 human chondrosarcoma cells.

However, the essential cellular and molecular targets and a signaling mechanism for resveratrol have not been completely defined. Although type II collagen and sulfated proteoglycan are important for differentiation and COX-2 is significant in the inflammatory response, the underlying regulatory mechanisms of type II collagen and COX-2 in articular chondrocytes are not yet understood. In the present study, the effects of resveratrol on the expression of type II collagen and COX-2 in rabbit articular chondrocytes were investigated and the regulatory mechanisms involved in these effects were investigated.

Resveratrol induces apoptosis or anti-proliferative effects in a variety of cell types, including prostate, breast, lung, leukemia, bladder and ovarian cancer cells (33). It prevents the proliferation of tumor cells by inhibiting DNA synthesis and cell cycle progression and by modulating a series of signaling molecules (17). Resveratrol reduces cell apoptosis and the inflammatory response induced by inflammatory cytokines and inhibits dedifferentiation in arthritic chondrocytes (30,34). In the present study, it was found that resveratrol inhibited the proliferation of rabbit articular chondrocytes (data not shown).

Studies have identified that the diverse effects of resveratrol are regulated differentially according to numerous conditions, such as the concentration of resveratrol and the treatment period (16–18). At a higher dose, resveratrol is pro-apoptotic, inducing apoptosis in cancer cells by exerting a death signal. In addition, at a higher dose, resveratrol depresses cardiac function, elevates the levels of apoptotic protein expression, which results in an unstable redox environment, and increases myocardial infarct size and the number of apoptotic cells (17). The expression levels of proteins associated with cell survival are increased, which results in anti-apoptotic effects, when cells are treated with a low dose of resveratrol (16). Studies have indicated that it may be possible to use resveratrol to prevent and treat OA. For example, Shakibaei *et al* characterized the effects of IL-1β-induced suppression of collagen type II and β1-integrin signal receptor synthesis, and observed that the activation of caspase-3 and PARP cleavage were blocked by resveratrol (35,36). A study has suggested that resveratrol directly blocks caspase-3 and the subsequent cleavage of PARP and reverses the IL-1β-induced upregulation of ROS in chondrocytes (35). Furthermore, resveratrol inhibits the activation of NF-κB and thus downregulates NF-κB-regulated pro-inflammatory gene products such as COX-2, IL-1β and IL-6, which are important in the pathogenesis of OA (37). In the present study, it was demonstrated that a low concentration of resveratrol promotes differentiation, but treatment with a high concentration of resveratrol results in inducement of dedifferentiation (Figs. 1A and 2A), and resveratrol significantly induces the expression of type II collagen in a time-dependent manner (Figs. 1C and 2B). Treatment of rabbit articular chondrocytes with resveratrol was shown to induce the expression of COX-2 and increase PGE_2_ production in an dose-dependent manner, and the highest expression levels of COX-2 and PGE_2_ production were observed at 3 h after treatment with resveratrol (Figs. 3 and 4).

MAPK cascades have been shown to be key in the transduction of extracellular signals to cell responses. The MAPK signaling pathways relay, amplify and integrate signals from a wide range of stimuli prior to eliciting an appropriate physiological response that may include cell growth, proliferation, differentiation, development, inflammatory responses, apoptosis and invasion in mammalian cells (38,39). The PI3K/Akt signaling pathway is important for cell growth, differentiation and survival (40). In previous studies, chondrocyte differentiation and the inflammatory response were demonstrated to be associated with the MAPK and PI3K/Akt signaling pathways (41,29). Although the precise mode of resveratrol action has not yet elucidated, a few signaling pathways and molecular targets have been suggested. In several types of tumor cell line, resveratrol has inhibited the activation of JNK and its upstream MAPK/ERK and MEK (14). It has been reported that apoptosis through activation of p53, which is one of the chemotherapeutic effects of resveratrol, occurs through ERK/p38 (42). In addition, studies have demonstrated that resveratrol inhibits IL-1β-induced expression of COX-2 and production of PGE_2_, causing inhibition of the expression of cartilage-specific collagen type II (43,44). Resveratrol has been shown to induce apoptotic cell death, and suppression of pro-survival PI-3K/Akt signaling may be an important mediator in this process (25). In the present study, resveratrol activated all the ERK, p38, JNK and Akt signaling pathways that belong to the MAPK signaling system (Fig. 5). Therefore, in order to elucidate the association of these signaling systems with cell differentiation and the inflammatory response due to resveratrol, the ERK, p38, JNK and Akt signal transduction pathways were attenuated with their respective inhibitors, PD, SB, SP, TB and LY, following which the expression levels of type II collagen and COX-2 and the synthesized levels of proteoglycan and PGE_2_ were observed. The results showed that, with the exception of SP treatment, the increased type II collagen and COX-2 expression levels and increased levels of proteoglycan and PGE_2_ were attenuated following treatment with PD, SB, TB and LY (Figs. 6 and 7).

These results suggest that the differentiation and inflammatory response induced by resveratrol in rabbit articular chondrocytes are regulated through the ERK, p38 and Akt signaling pathways. Since various signaling pathways in addition to the MAPK and Akt signaling pathways, such as the PKC pathway, are associated with the regulation of intrachondrocytic reactions, further detailed studies are required. In addition, as the mechanisms behind the dedifferentiation induced by treatment with a high resveratrol concentration and the suppressed inflammatory response following exposure to resveratrol for long time periods are not known, these also require further investigation. Such study results may be used as fundamental data for the therapy of chondrocytic illnesses such as arthritis.
